# Efficacy and safety of clinically managed weight loss programs: a systematic review and meta-analysis protocol

**DOI:** 10.1186/s13643-021-01750-1

**Published:** 2021-07-02

**Authors:** Katrina Cachero, Matthew Granger, Rebecca C. Mollard, Nicole Askin, George N. Okoli, Ahmed M. Abou-Setta, Dylan MacKay

**Affiliations:** 1 Department of Food and Human Nutritional Sciences, Faculty of Agriculture and Food Sciences, University of Manitoba, Winnipeg, Manitoba Canada; 2 George & Fay Yee Centre for Healthcare Innovation, Max Rady College of Medicine, Rady Faculty of Health Sciences, University of Manitoba, 753 McDermot Avenue, Winnipeg, Manitoba R3E 0T6 Canada; 3 Chronic Disease Innovation Center, Seven Oaks Hospital, Winnipeg, Manitoba Canada; 4 University of Manitoba Libraries, University of Manitoba, Winnipeg, Manitoba Canada; 5 Department of Community Health Sciences, Max Rady College of Medicine, Rady Faculty of Health Sciences, University of Manitoba, Winnipeg, Manitoba Canada

**Keywords:** Obesity, Weight loss program, Dietitian, Exercise, Psychology, Physician, Nurse practitioner

## Abstract

**Background:**

Obesity has become a major driver in the burden of chronic diseases. The Canadian Clinical Practice Guidelines recommend a lifestyle intervention for the management and prevention of obesity. This includes behavior modification, dietary counseling, and physical activity. With the market overwhelmed with weight loss programs, the majority are focused on low-calorie diets and general recommendations for exercise. Most are not personalized and are not administered by healthcare professionals. An interdisciplinary team of highly trained healthcare professionals has the ability to provide medically sound and safe advice in all aspects of an individuals’ life, such as lifestyle, sleep, mental health, and behaviors. A clinically managed weight loss program is defined as a team including a dietitian, exercise professional, psychologist, and/or physician or nurse practitioner oversight. With limiting results in the literature regarding clinically managed weight loss programs, it is difficult to conclude whether it may be effective. Therefore, the objective of this systematic review is to assess clinically managed weight loss programs, with a physician or nurse practitioner oversight in comparison with non-clinically managed weight loss programs with no physician oversight or nurse practitioner oversight in adults who are living with overweight or obesity.

**Methods:**

A literature search will be executed by a knowledge synthesis librarian on MEDLINE, Cochrane Central, Embase, PsycINFO, and CINAHL. The data collected will be extracted, stored, and managed in MS Excel 2016. The extraction of the data will include study details, study population details, health team details, intervention details, and outcome details.

**Discussion:**

The prevalence of obesity has been increasing throughout the decades. The results from this systematic review may aid in recommending a more clinically safe weight loss program for those who struggle with overweight or obesity.

**Systematic review registration:**

PROSPERO CRD42020170014

**Supplementary Information:**

The online version contains supplementary material available at 10.1186/s13643-021-01750-1.

## Background

The prevalence of adults who are overweight or with obesity has been increasing throughout the past three decades with almost two-thirds of Canadian adults being overweight or with obesity [1, 2]. This increasing prevalence is believed to be a major driver in the burden of chronic diseases [3]. The World Health Organization defines overweight and obesity as abnormal or excessive fat accumulation that may impair health [4]. There are different ways to measure overweight or obesity with the most common measurement being body mass index (BMI). Individuals with a BMI over 25 are classified as overweight and those over 30 are classified with obesity. The current Canadian Clinical Practice Guidelines (CCPG) on the management and prevention of obesity in adults recommend a comprehensive lifestyle intervention including behavior modification, dietary counseling, and physical activity as the first-line treatment option to achieve clinically significant weight loss [5]. Weight reduction is well documented to improve cardiovascular risk factors (such as blood pressure, low-density lipoprotein cholesterol, and triglycerides) and blood glucose metabolism in individuals who are overweight or with obesity [5, 6].

Weight loss programs are primarily focused on low-calorie diets and rarely include medical oversight. Weight loss programs such as Weight Watchers, Jenny Craig, or Nutrisystems have nutrition, physical activity, and behavioral strategy components but are not personalized to the individual or administered by healthcare professionals [7]. Based on the 2006 CCPG, a weight management program should involve a nutrition health professional, an exercise professional, and a clinical psychologist [5]. With this type of interdisciplinary team, all aspects of an individuals’ life are considered (i.e., lifestyle, sleep, mental health, behaviors). A weight loss program which is directed by dietitians, exercise professional, and/or psychologist, with physician or nurse practitioner (prescriber) oversight, is considered a clinically managed weight loss program. Clinicians are able to actively monitor a participants’ health and potentially adjust medications throughout the weight loss program. A study by Tapsell and Neale [8] found that an interdisciplinary intervention with physician oversight produced greater and more clinically significant weight loss. Additionally, interdisciplinary weight loss programs have shown improvement in other areas other than weight, such as eating behaviors, lipid profiles, aerobic capacity, and overall quality of life [8–10].

The consumer marketplace is overwhelmed with weight loss programs, with the majority being focused on calorie-reduced diets [7]. Not all of these programs include exercise, and most do not include physician oversight and may not be customizable. Clinician oversight may provide an additional benefit because clinicians are highly trained professionals and have the ability to prescribe or adjust medications and provide medically sound and safe advice. However, some potential drawbacks of physician oversight to a weight loss program’s success may include the added expense, participant stress, or feelings of judgment, and with the increased number of healthcare professional involvement, there may be hierarchy conflict.

With varying results in the literature, it is difficult to conclude whether clinician oversight in weight loss programs is more effective or not. Therefore, the objective of this systematic review is to assess the efficacy of clinically managed weight loss programs, with a physician or nurse practitioner oversight, in comparison with non-clinically managed weight loss programs with no physician or nurse practitioner oversight in adults who are overweight or with obesity.

## Research question

Do weight loss programs in adults who are overweight or with obesity directed by dietitians, exercise professionals, and/or psychologists, with a physician or nurse practitioner oversight, lead to greater program success compared to similar programs without physicians or nurse practitioners?

## Methods

### Study selection

A literature search strategy for MEDLINE will be designed by a knowledge synthesis librarian and peer reviewed by a second, independent librarian using the PRESS checklist [11]. The peer-reviewed search strategy will then be adapted for other bibliographic databases (Cochrane Central, Embase, PsycINFO, and CINAHL) and executed by a knowledge synthesis librarian. Identified citations from the executed searches will be screened for eligibility by two independent systematic reviewers on Rayyan (Rayyan, Doha, Qatar) [12]. The number of ineligible citations at the title/abstract screening stage will be recorded, and both the number and the reason for ineligibility will be recorded at the full-text article screening stage. Any disagreements during these screening stages will be resolved by discussion between the two systematic reviewers with a third reviewer to adjudicate, if necessary.

### Eligibility criteria

The following studies will be included:

1. Population: overweight or with obesity (BMI > 25) adults (18–65 years of age) from North America, Europe, Australia, and New Zealand (≥ 80% of the trial population).
2. Intervention: clinically managed weight loss programs with physician or nurse practitioner oversight.
3. Comparator: weight loss programs with no physician or nurse practitioner oversight.
4. Outcomes:
  - Primary: weight.
  - Secondary: BMI, waist circumference, body fat percentage, lipid profile, blood pressure, adherence to program, withdrawal from program, and quality of life.
  - Safety: any reported adverse events.
5. Study design: randomized controlled trials (parallel or cluster-design). For cross-over trials, we will use the data before the cross-over.
6. Publications from the year 1990 to the date of search.
7. Full-text manuscript in the English language (for feasibility).

The cost of living, access to food options, and healthcare systems around the world differ markedly; countries with characteristics similar to the Canadian setting were selected, and this includes North America, Europe, Australia, and New Zealand.

### Data extraction

We will utilize data extraction forms developed in MS Excel 2016 (Microsoft Corporation, Redmond, WA, USA) [13] and piloted on a small selection of studies for quality assurance. Extracted data will be stored and managed in MS Excel. Two systematic reviewers will independently extract data from included studies. Any disagreements will be resolved by a discussion between the two reviewers, and a third reviewer will adjudicate if necessary. The following data will be extracted from the included studies:

*Study details*: name of the first author, the year the study was conducted, year of publication, country, setting, population demography, study size, and funding source.

*Study population details*: type of population (for example, adults), age, sex distribution, and health and socioeconomic status.

*Health team details*: profession.

*Intervention details*: name, type, method of intervention, measure (amount/extent), duration, and contact hours.

*Outcome details*: (see above) data will be extracted at the end of the trial and at the longest reported follow-up.

### Assessment of risk of bias

We will assess the risk of bias using the Cochrane Risk of Bias Tool 2.0 [14]. This tool assigns a judgment of high, some concerns, and low risk of bias for each of the following domains: bias arising from the randomization process, bias due to deviations from intended interventions, bias due to missing outcome data, bias in measurement of the outcome, and bias in selection of the reported result. Any disagreements will be resolved by a discussion between the two reviewers or by involving a third reviewer if necessary.

### Data analysis

We will conduct a meta-analysis where feasible, using a random effects model implemented in RevMan (version 5.3.5) [15]. We will express pooled continuous data as mean differences or as standardized mean differences where measures of the same outcome are with different scales, presenting the 95% confidence intervals. Pooled dichotomous data will be presented as a risk ratio or, for rare outcomes, using the Peto odds ratio. We will assess and quantify the statistical heterogeneity between the included studies using the I-squared statistic (I^2^). We will assess for publication bias visually using funnel plots of the effect size versus sample size for each included study and using Egger’s regression test.

Then, a priori subgroup and sensitivity analyses are proposed depending on the number of studies included and the availability of data: differences between low risk of bias and some concerns/high risk of bias studies, intervention types, clinician type, population type, participant sex, comorbidity status, and geographical location (for example, continent).

### Study outcome dissemination

In addition to a peer-reviewed academic publication, we will present our findings at appropriate academic meetings.

## Supplementary Information

**Additional file 1:** MEDLINE Preliminary Search Strategy.

## Data Availability

No datasets have been generated related to this published article.

## Abbreviations

BMI: Body mass index
CCPG: Canadian Clinical Practice Guidelines
CDIC: Chronic Disease Innovation Centre
WI: Wellness Institute
SOGH: Seven Oaks General Hospital
