# Supplementary material for: Efficacy and safety of clinically managed weight loss programs: a systematic review and meta-analysis protocol
Source: Syst Rev. 2021 Jul 2;10:197. doi: 10.1186/s13643-021-01750-1 (PMC8254293; doi:10.1186/s13643-021-01750-1)
Supplement: Supplementary file 1 — Additional file 1: MEDLINE Preliminary Search Strategy. [file 13643_2021_1750_MOESM1_ESM.docx]

**MEDLINE Preliminary Search Strategy**

1. exp overnutrition/dh, dt, pc, th or exp body weight/dh, dt, pc, th

2. exp weight reduction programs/ or weight loss/ or exp diet, carbohydrate-restricted/ or diet, fat-restricted/ or diet, paleolithic/ or diet, reducing/ or caloric restriction/ or exp anti-obesity agents/

3. (exp exercise/ or exp exercise therapy/ or "physical education and training"/ or exp diet/ or exp diet therapy/) and (exp overnutrition/ or exp body weight/)

4. ((weight or obesity) adj3 (loss or reduc* or program* or manag* or interven*)).ti,ab,kf

5. (((diet* or calori*) adj3 (reduc* or restrict* or therap*)) and (weight or overweight or obesity or obese)).ti,ab,kf

6. ((weight or overweight or obese or obesity) adj10 (lifestyle or behavi* therap* or cogniti* therap* or counsel* or exercis* or kinesiotherap* or physical* activ* or fitness)).ti,ab,kf

7. ((low-calorie or low-fat or fat-free or low-carb* or keto* or paleo* or atkins or caveman or stone age or hunter-gatherer) adj3 diet*).ti,ab,kf

8. (antiobesity or anti-obesity or anorectic* or anorexic drug* or anorexigenic* or (appetite adj2 (suppress* or depress* or repress* or control*))).ti,ab,kf

9. or/1-8

10. exp physicians/ or exp professional role/ or exp nurse practitioners/ or nurse clinicians/

11. ((physician* or doctor* or gastroenterologist* or bariatrician* or clinician* or gp or general practitioner* or internist* or hospitalist* or allergist* or cardiologist* or endocrinologist* or geriatrician* or gerontologist* or obstetrician* or gynecologist* or registrar* or dermatologist* or nephrologist* or neurologist* or oncologist* or ophthalmologist* or otolaryngologist* or neonatologist* or pulmonologist* or rheumatologist* or surgeon* or urologist* or hematologist* or haematologist* or hepatologist* or immunologist* or intensivist* or nurse practitioner* or nurse clinician* or np or apn or advance* practice nurse* or nurse specialist* or primary care provider*) adj3 (led or lead or leading or driven or administer* or manag* or guid* or oversight* or oversee* or deliver* or direct* or perform* or aegis or control* or conducted or govern* or supervis* or implement* or involv* or integrat* or role*)).ti,ab,kf

12. or/10-11

13. 9 and 12

14. 13 not ((exp animals/ not humans.sh.) or ((exp child/ or exp infant/ or adolescent/) not exp adult/))

15. Randomized Controlled Trials as Topic/

16. randomized controlled trial/

17. Random Allocation/

18. Double Blind Method/

19. Single Blind Method/

20. clinical trial/

21. clinical trial, phase i.pt

22. clinical trial, phase ii.pt

23. clinical trial, phase iii.pt

24. clinical trial, phase iv.pt

25. controlled clinical trial.pt

26. multicenter study.pt

27. (pragmatic clinical trial or randomized controlled trial).pt

28. Clinical Trials as topic/

29. (clinical adj trial$).ti,ab,kf

30. (RCT or RCTs or quasi-random* or quasi-experimental*).ti,ab,kf

31. ((singl* or doubl* or trebl* or tripl*) adj (blind* or dumm* or mask*)).ti,ab,kf

32. PLACEBOS/

33. (placebo$ or sham).ti,ab,kf

34. (randomized or randomised or randomly).ab

35. trial.ti

36. or/15-35

37. 14 and 36

38. limit 37 to yr="1990 -Current"
